# Virus Detection and Monitoring of Viral Load in Crimean-Congo Hemorrhagic Fever Virus Patients

**DOI:** 10.3201/eid1307.070068

**Published:** 2007-07

**Authors:** Roman Wölfel, Janusz T. Paweska, Nadine Petersen, Antoinette A. Grobbelaar, Patricia A. Leman, Roger Hewson, Marie-Claude Georges-Courbot, Anna Papa, Stephan Günther, Christian Drosten

**Affiliations:** * Bundeswehr Institute of Microbiology, Munich, Germany; † Bernhard Nocht Institute for Tropical Medicine, Hamburg, Germany; ‡ National Institute for Communicable Diseases, Sandringham, South Africa; § Health Protection Agency, Porton Down, Salisbury, United Kingdom; ¶ Institute Pasteur, Lyon, France; # Aristotle University of Thessaloniki, Thessaloniki, Greece

**Keywords:** Crimean-Congo hemorrhagic fever virus, RT-PCR, 5′-nuclease assay, dispatch

## Abstract

We developed a real-time reverse transcription–-PCR that detected 1,164 copies/mL of Crimean-Congo hemorrhagic fever virus per milliliter of serum at 95% probability (probit analysis) and was 100% concordant with nested PCR on 63 samples from 31 patients with confirmed infection. Infected patients who died appeared to have higher viral loads; low viral loads correlated with IgG detection.

Crimean-Congo hemorrhagic fever (CCHF) is a tickborne viral zoonosis that occurs widely in Africa, Asia, and Eastern Europe. It is caused by *CCHF virus* (CCHFV), a segmented, negative-stranded RNA virus belonging to the family *Bunyaviridae*, genus *Nairovirus*. CCHF has a fatality rate of ≈30% and a potential for nosocomial spread (*1*). Early diagnosis of CCHF is important for case management and protection of medical staff.

Diagnostic assays for CCHF include virus culture, antigen-detection enzyme immunoassay (EIA), antibody-detection EIA, and reverse transcription–PCR (RT-PCR) (*2*). Virus detection is the main diagnostic method in the acute stage of disease, and RT-PCR is most sensitive method of detection. However, because of the remarkable genetic variability among CCHFV strains, all current RT-PCRs either lack sensitivity or focus on the detection of local CCHFV variants only (*3*–*6*).

We describe the first real-time RT-PCR that rapidly and reliably detects the global spectrum of clinically relevant virus strains. An extended strategy of probe design was implemented to cover such high variability. Sensitivity was demonstrated by testing virus strain collections from several different Biosafety Level 4 laboratories, essentially covering the full range of global diversity of CCHFV (Figure 1). A comprehensive panel of original clinical samples from persons with confirmed cases of CCHF was used for clinical evaluation; the samples were collected by World Health Organization reference facilities.

**Figure 1:**
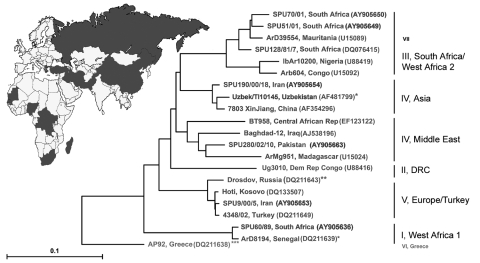
Global distribution and phylogenetic relationships of Crimean-Congo hemorrhagic fever virus (CCHFV) strains selected for design and validation of the assay. All strains except those marked with asterisks were tested. Phylogenetic analysis was based on available 450-bp sequences (from the National Center for Biotechnology Information) of CCHFV small (S-) segment and generated by the neighbor-joining method with TreeCon for Windows (version 1.3b; Yves van de Peer, University Konstanz, Germany). Nomenclature of CCHFV clades is based on (*7*). Note that group VII can be resolved only when analyzing the M-segment, not the s-segment as shown here. *These CCHFV strains are shown for reference, but they were not available for testing. **This strain was not available; however, strain Kosovo, which is almost identical, was tested instead. ***Strain AP92 has also not been available for testing. It was isolated from a *Rhipicephalus bursa* tick and has never been associated with human disease.

## The Study

**Figure 2:**
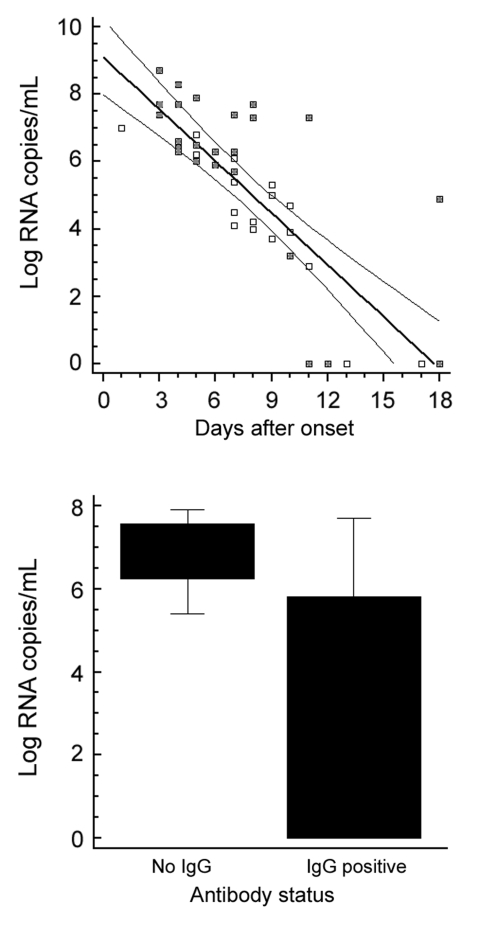
Clinical evaluation. Upper panel: plasma viral load over time in 44 samples from 17 patients. Samples from patients who died are marked with a filled square. Lower panel: plasma viral load in relation to antibody status in 16 samples with and 27 samples without detectable immunoglobulin G (IgG) antibodies. Only IgG status was taken as reference because only 2 patients had IgM without IgG. The difference of means between the 2 groups was highly significant (*t* test, p = 0.00005). Boxes indicate 25th through 75th percentiles; error bars indicate 5th and 95th percentiles. A) Oligonucleotides. The Figure 1 shows oligonucleotide binding sites from representative strains taken from the full CCHFV alignment used for assay design. The full alignment can be accessed at www.bni-hamburg.de. All sequences show the plus (coding) sense cDNA (i.e., the reverse complement of virus RNA). The forward primer RWCF and probes SE01 and SE03 are located on the plus strand. The reverse primer RWCR and probe SE0A are located on the minus strand. B) Analytical evaluation. Left and middle panel: amplification of a representative set of virus RNA (strain identity in legend) with probe SE01 only (left panel) and with the full set of 3 probes (middle). Note that all strains were detected with probe SE01 alone, but some strains showed weak signal. The additional probes increased the signal for such weak strains without lowering the signal for others. The 2 completely flat lines on both panels are negative controls. Left panel: probit analysis performed with the final assay protocol. The x*-*axis shows the calculated input concentration of synthetic virus RNA of strain BT-958 (EF123122), the y*-*axis shows the observed hit rates in 5 replicate reactions per concentration and the calculated detection probabilities according to the dose-response model. Upper and lower curves represent 95% confidence limits of the model. C) Clinical evaluation. Left panel: plasma viral load over time in 44 samples from 17 patients. Samples from patients who died are marked with a filled square. Right panel: plasma viral load in relation to antibody status in 16 samples with and 27 samples without detectable IgG antibodies. Only IgG status was taken as reference because only 2 patients had IgM without IgG. The difference of means between the 2 groups was highly significant (*t* test, p = 0.00005). Boxes indicate 25th through 75th percentiles; error bars indicate 5th and 95th percentiles. CCHFV, Crimean-Congo hemorrhagic fever virus.

Primers and probes were selected on the basis of an alignment of S segment sequences of 61 CCHFV isolates from all known CCHF-endemic regions worldwide (*7*) (representative sequences shown in expanded online version of Figure 2. Oligonucleotide melting points, folding characteristics, and cross-hybridization properties were determined by using the Primer Express software package (Applied Biosystems; Foster City, CA, USA). Primers were selected to amplify a 181-bp region near the 5′-end of the S segment. The capability of these primers to amplify 12 representative CCHFV strains from distinct CCHF-endemic regions was confirmed initially by gel detection RT-PCR (data not shown) (Figure 2, panel B).

For real-time PCR, identifying a simple detection probe compatible with all known CCHFV strains was not possible. Therefore, a broad-range probe was formulated on the basis of the observation that the non–Watson/Crick base pair G:T is almost as thermodynamically stable as regular Watson/Crick base pairs, whereas A:C is very unstable (*8*). Thus, the probe was placed on the DNA strand that provided more G:T mismatches than complementary A:C mismatches, and the resulting G:T mismatches were not compensated for. As shown in the left panel of Figure 2B this probe, designated SE01, detected all 12 representative strains. Because signal intensity varied according to the strain detected, a strain that provided low signal (BT956, Figure 2B) was chosen for evaluation of sensitivity. Its full S segment RNA was cloned and transcribed in vitro to obtain a quantitative RNA standard (*9*). Cloning, in vitro transcription, purification, and quantification were performed as previously described (*10*). End-point dilution showed that single copies of RNA could be detected despite low overall fluorescence (data not shown). Nevertheless, variation in signal intensity between strains was adjusted by the following 2 modifications. First, an additional oligonucleotide (SE03) was introduced at the same binding site as SE01. This probe had 2 effects: first, a pyrimidine base (IUB-code “Y,” 50% C and 50% T) was generated at 2 positions of balanced C/T polymorphisms. Second, a “keto” base (IUB-code “K,” 50% G and 50% T) resulted at 1 position of total variability (A, C, G, T). RNA from the 12 representative strains was tested, and those strains that still provided low signal were realigned separately. On the basis of the second alignment, an additional probe was selected at an alternative binding site to prevent interference with probes at the first binding site. It was placed on the minus strand to obtain more G:T mismatches than complementary A:C mismatches (see above). The improvement obtained by the additional probes on the set of representative strains is shown in Figure 2B, middle panel. The final assay protocol is summarized in the Table.

**Table:** Protocol for real-time reverse transcription–PCR

Oligonucleotide*	Purpose, concentration in nM	Sequence and label (5′→3′)	Position (U88410)†
RWCF	Forward primer, 600	CAAGGGGTACCAAGAAAATGAAGAAGGC	1068–1095
RWCR	Reverse primer, 600	GCCACAGGGATTGTTCCAAAGCAGAC	1248–1223
SE01	Broad-range probe, 100	FAM-ATCTACATGCACCCTGCTGTGTTGACA-TAMRA	1172–1198
SE03	Additional probe, 100	FAM-ATTTACATGCACCCTGCCGTGCTTACA-TAMRA	1172–1198
SE0A	Additional probe, 100	FAM-AGCTTCTTCCCCCACTTCATTGGAGT -TAMRA	1131–1106

*All oligonucleotides were used in an assay with the following protocol: 25-µL reaction volume, 5-µL plasma RNA (QIAamp Viral RNA mini kit; QIAGEN, Valencia, CA, USA), 1× concentration of buffer and enzymes from the OneStep RT-PCR kit (QIAGEN), and 400 µmol dNTP, 800-ng nonacetylated bovine serum albumin (Sigma-Aldrich, Munich, Germany). The cycling parameters followed in a Roche LightCycler 1.2 (Roche, Penzberg, Germany) were as follows: 30 min at 50°C, 15 min at 95°C, 46× 15 s at 94°C, and 30 s at 59°C. Fluorescence acquisition occurred at the 59°C step, wavelength filter F1/F2 mode. †GenBank accession number.

For precise evaluation of analytical sensitivity, a series of human plasma samples was spiked with the RNA standard from strain BT-958 in concentrations ranging from 100,000 to 10 copies per mL. Testing was done on 5 replicate reactions per concentration, and probit analysis was conducted as shown in the expanded online version of Figure 2, panel B, right graph (*11*). The calculated limit of detection, defined as the concentration down to which >95% of conducted tests can be expected to be positive, was 13.6 copies per reaction (p = 0.05). This corresponded to 1,164 copies per mL of plasma (95% confidence interval, 780–2990 copies/mL).

Cross-reactivity was excluded by testing DNA or RNA from cultures or high-titered clinical samples containing Dugbe virus, Rift Valley fever virus, Sudan Ebolavirus Gulu, Lassa virus AV, yellow fever virus, dengue virus types 1–4, Japanese encephalitis virus, West Nile virus Uganda, Venezuelan equine encephalitis virus, Sindbis virus, Ross River virus, Epstein-Barr virus, hepatitis C virus, human cytomegalovirus, monkeypox virus, poliomyelitis virus types 1–3, rabies virus RSDD, *Bacillus anthracis*, *Leptospira interrogans*, *Listeria monocytogenes*, *Neisseria meningitidis*, *Coxiella burnetii, Rickettsia prowazekii, R. rickettsii,* and *Plasmodium falciparum*. An additional 128 blood specimens collected during the course of the study from 128 patients with conditions other than CCHF all tested negative for CCHF virus.

The real-time RT-PCR was used to test and quantify 63 serum samples from 31 patients with laboratory-confirmed CCHFV infection; the samples were obtained 1–18 days after symptom onset. All samples had nested RT-PCR results positive for CCHFV (*3*), and all were also positive by the new real-time RT-PCR. For 21 patients with confirmed CCHF (17 from South Africa, 3 from Iran, and 1 from Pakistan), viral load was quantified and compared with other standard diagnostic methods for CCHFV detection (Appendix Table). Again, sensitivity of the new assay was at least as high as that of nested PCR. As shown in Figure 2, there was a clear correlation between viral load and duration of symptoms in these patients. Clinical outcome could not be correlated clearly with viral load, although patients who died of the disease seemed, in general, to have higher viral loads (Figure 2, filled squares). The appearance of antibodies correlated clearly with lower viral loads (Figure 2).

## Conclusions

To our knowledge, this is the first PCR validated with representative CCHFV strains from nearly all regions worldwide where the virus is endemic. High sensitivity enables reliable detection of virus in early stages of the infection, when antibody detection is unreliable or impossible. By eliminating the need for postamplification product processing, real-time RT-PCR enables shortened turnaround times for reporting results, which is critical for deciding on isolation and contact-tracing for suspected case-patients. Quantification of viral load may assist in estimating the patient’s infectivity. It may also assist in predicting the clinical outcome and could be used to monitor viral load in patients receiving ribavirin treatment (*12*). Our study provides baseline data on CCHF viral load throughout the acute stage of the illness. High viral load tended to indicate fatal outcome, and lower viral load was generally associated with detectable antibodies. Because detectable antibody response correlates with good outcome (*13*), viral load will probably be a useful predictor of clinical progress. These preliminary data are highly encouraging for further studies on larger patient cohorts.

## Supplementary Material

Appendix Table Comparison of 4 standard diagnostic methods with the novel quantitative real-time reverse transcriptase-PCR (qPCR) assay, using primary specimens and quantification of Crimean-Congo hemorrhagic fever viral load*
