# Supplementary material for: Virus Detection and Monitoring of Viral Load in Crimean-Congo Hemorrhagic Fever Virus Patients
Source: Emerg Infect Dis. 2007 Jul;13(7):1097–100. doi: 10.3201/eid1307.070068 (PMC2878241; doi:10.3201/eid1307.070068)
Supplement: Appendix Table — Comparison of 4 standard diagnostic methods with the novel quantitative real-time reverse transcriptase-PCR (qPCR) assay, using primary specimens and quantification of Crimean-Congo hemorrhagic fever viral load* [file 07-0068_appT-s1.pdf]

Appendix Table. Comparison of 4 standard diagnostic methods with the novel quantitative real-time reverse transcriptase–PCR (qPCR) assay, using primary specimens and quantification of Crimean-Congo hemorrhagic fever viral load*

Patient, age in years (year sample collected, patient outcome)†	No. days after disease onset	Mouse brain inoculation‡	Vero cell culture‡	Antibodies§	Conventional PCR¶	Novel qPCR viral copies (log/mL)
JN, 47 (2001, survived)	4	Pos	Pos	Neg	Pos	6.6
	7	Pos	Pos	Pos (IgM only)	Pos	6.1
	9	Pos	ND	Pos	ND	5.0
	10	Neg	ND	Pos	ND	4,7
	13	ND	ND	Pos	ND	Neg
	17	ND	ND	Pos	ND	Neg
WK, 33 (2001, survived)	5	Pos	ND	Neg	Pos	6.8
	7	Neg	ND	Pos	ND	4.1
	8	Neg	ND	Pos	ND	4.0
	10	Neg	ND	Pos	ND	3.9
	11	Neg	ND	Pos	ND	2.9
	12	Neg	ND	Pos	ND	Neg
EH, 46 (2001, died)	5	Pos	Pos	Pos (IgM only)	Pos	6.2
LK, 54 (2001, died)	3	Pos	Pos	Neg	Pos	7.7
JNM, 33 (2002, survived)	1	Pos	Pos	Neg	Pos	7.0
	5	Pos	ND	Pos	ND	6.0
	7	Pos	Pos	Pos	ND	4.5
	8	Neg	ND	Pos	ND	4.2
	9	Neg	ND	Pos	ND	3.7
	13	ND	ND	Pos	ND	Neg
FF, 67 (2004, died)	7	Pos	ND	Neg	Pos	5.7
ME, 51 (2006, died)	3	Neg	ND	ND	ND	>8.7
	4	Neg	ND	ND	ND	8.3
	5	Pos	ND	ND	ND	6.5
	6	Neg	ND	Neg	Pos	6.3
	7	Pos	ND	ND	ND	6.3
	8	Pos	ND	ND	ND	7.3
JVR, 33 (2006, died)	4	Pos	ND	Neg	ND	6.3
AM, 25 (2006, died)	10	Pos	ND	Pos	Pos#	3.2
	12	ND	ND	Pos	ND	Neg
	18	ND	ND	Pos	ND	Neg
NVDM, 53 (2001, died)	6	Pos	Pos	Pos	Pos	5.9
RDT, 44 (2001, survived)	7	Pos	Pos	Pos	Pos	5.4
PK, 65 (2002, died)	3	Pos	Pos	Neg	Pos	7.4
	4	Pos	Pos	Neg	Pos	7.7
PJM, 49 (2002, died)	7	Pos	Pos	Pos	Pos	7.4
	8	Pos	ND	Pos	ND	7.7
WZ, 69 (2004, died)	4	Pos	Pos	Pos	Pos	6.4
	11	Neg	ND	Pos	ND	Neg
PTK, 35 (2004, died)	11	Pos	Pos	Pos	Pos	7.3
	18	Pos	ND	Pos	ND	4.9
RR, 41 (2004, died)	4	Neg	Neg	Neg	Pos	7.7
	5	Neg	ND	Neg	Pos	7.9
JJB, 58 (2004, survived)	9	Pos	ND	Pos	Pos	5.3
J, 55 (2002, unknown)	Unknown	Neg	ND	Neg	Pos	5.4
KD, 45 (2002, unknown)	Unknown	Neg	ND	Pos	Pos	5.8
AZ, 80 (2002, unknown)	Unknown	Neg	ND	Neg	Pos	7.1
U, 45 (2002, unknown)	Unknown	Pos	ND	Neg	Pos	6.3

*Neg = negative test result; Pos = positive test result; ND = not done.
†All patients except ME were male.
‡As described in Shepherd et al. (1).

§Indirect immunofluorescence assay; >1:80 for immunoglobulin G (IgG) and IgM is positive, as described in Swanepoel et al. (2).
¶Burt et al. (3).
#Only in nested PCR.
